# Steroidogenic Acute Regulatory Protein Overexpression Correlates with Protein Kinase A Activation in Adrenocortical Adenoma

**DOI:** 10.1371/journal.pone.0162606

**Published:** 2016-09-08

**Authors:** Weiwei Zhou, Luming Wu, Jing Xie, Tingwei Su, Lei Jiang, Yiran Jiang, Yanan Cao, Jianmin Liu, Guang Ning, Weiqing Wang

**Affiliations:** 1 Shanghai National Clinical Center for Endocrine and Metabolic Diseases, Shanghai Key Laboratory for Endocrine Tumors, Department of Endocrine and Metabolic Diseases, Rui-Jin Hospital, Shanghai Jiao-Tong University School of Medicine, Shanghai, China; 2 Department of Pathology, Rui-Jin Hospital, Shanghai Jiao-Tong University School of Medicine, Shanghai, China; 3 Laboratory of Endocrinology and Metabolism, Institute of Health Sciences, Shanghai Institutes for Biological Sciences, Chinese Academy of Sciences, Shanghai, China; Harvard Medical School, UNITED STATES

## Abstract

The association of pathological features of cortisol-producing adrenocortical adenomas (ACAs) with somatic driver mutations and their molecular classification remain unclear. In this study, we explored the association between steroidogenic acute regulatory protein (StAR) expression and the driver mutations activating cyclic adenosine monophosphate (cAMP)/protein kinase A (PKA) signaling to identify the pathological markers of ACAs. Immunohistochemical staining for StAR and mutations in the protein kinase cAMP-activated catalytic subunit alpha (*PRKACA*), protein kinase cAMP-dependent type I regulatory subunit alpha (*PRKAR1A*) and guanine nucleotide binding protein, alpha stimulating (*GNAS*) genes were examined in 97 ACAs. The association of StAR expression with the clinical and mutational features of the ACAs was analyzed. ACAs with mutations in *PRKACA*, *GNAS*, and *PRKAR1A* showed strong immunopositive staining for StAR. The concordance between high StAR expression and mutations activating cAMP/PKA signaling in the ACAs was 99.0%. ACAs with high expression of StAR had significantly smaller tumor volume (*P* < 0.001) and higher urinary cortisol per tumor volume (*P* = 0.032) than those with low expression of StAR. Our findings suggest that immunohistochemical staining for StAR is a reliable pathological approach for the diagnosis and classification of ACAs with cAMP/PKA signaling-activating mutations.

## Introduction

Cortisol-producing adrenocortical adenomas (ACAs) are the main cause of adrenal Cushing’s syndrome, which leads to a variety of metabolic abnormalities including central obesity, diabetes, hypertension, myopathy, and osteoporosis [1,2]. Recent mechanistic investigations have demonstrated that aberrant cyclic adenosine monophosphate (cAMP)/protein kinase A (PKA) signaling results in the development of ACAs [3–7]. Whole exome sequencing of ACAs has identified a series of mutations in the *PRKACA*, *PRKAR1A*, and *GNAS* genes that result in PKA activation and cortisol overproduction [8–13]. Genetic sequencing and molecular classification of ACAs would contribute significantly to the clinical diagnosis of adrenal Cushing’s syndrome. However, the lack of reliable markers has restricted the development of rapid and cost-effective pathological approaches to the molecular diagnosis of ACAs.

Activating hotspot *PRKACA* mutations are dominant in ACAs [8–11], and inactivating *PRKAR1A* [14] and activating *GNAS* mutations [10,11] are also commonly identified. These mutational events lead to activation of cAMP/PKA signaling and increased phosphorylation of downstream targets including the cAMP response element-binding (CREB) and steroidogenic acute regulatory (StAR) proteins, which induce cortisol biosynthesis in the adrenal cortex [11]. Our previous study showed that an activating *PRKACA* mutation promoted PKA substrate phosphorylation and StAR activation [9]. Furthermore, RNA sequencing and western blot data revealed that the mRNA and protein levels of StAR were significantly higher in ACAs with mutations in *PRKACA* than in the wild type [9]. Therefore, we speculated that StAR might be a marker for activation of cAMP/PKA signaling in ACAs.

StAR is the first key mediator of steroidogenesis, which stimulates the initial mitochondrial metabolism of cholesterol to pregnenolone by enhancing the transfer of cholesterol from the outer membrane to the cytochrome P450 cholesterol side-chain cleavage enzyme (P450scc) in the inner membrane [15,16]. In *zona fasciculata* cells of the adrenal cortex, adrenocorticotropic hormone (ACTH) binds to the G-protein-coupled melanocortin-2 receptor, thereby activating adenylate cyclase through the Gs-α subunit and generating intracellular cAMP [6,8–13]. The cAMP then binds to the regulatory subunit of PKA to release the PKA catalytic subunit, which increases PKA activity and promotes the expression and phosphorylation of StAR [17]. Upregulation of StAR indicates enhanced production of cortisol in the adrenal cortex.

In the present study, we aimed to explore the association of StAR expression with the mutated genes in cAMP/PKA signaling and functional characteristics of the ACAs. To this end, genetic sequencing and immunohistochemical staining for StAR were performed in 97 ACAs. We believe that our findings may provide important clinical insights into ACAs with cAMP/PKA signaling-activating mutations.

## Materials and Methods

### Subject selection

We previously described 64 patients (exploratory cohort) with ACAs with identified genetic alterations [9], and subsequently validated an additional 33 patients (confirmatory cohort), who were diagnosed with autonomous unilateral cortisol-producing adenomas. The diagnosis of ACTH-independent Cushing’s syndrome was based on the results of hormonal investigations [18], whereas the pathological examination of the adrenal tissues confirmed the ACA diagnosis. No aldosterone-producing adrenal adenomas or adrenocortical carcinomas were included in the study.

### Hormonal assessment and biochemical methods

The systematic clinical and hormonal explorations were performed preoperatively. All of the patients underwent the same dexamethasone suppression test procedures: standard 2 mg/day (low-dose dexamethasone test, LDDST) and 8 mg/day suppression tests. The serum and urinary free cortisol were examined by using the Access Immunoassay Systems (Beckman Coulter Inc., Fullerton, CA, USA). The ACTH levels were determined by using an ELSA-ACTH immunoradiometric assay (Cisbio Bioassays, France). The original data of the computed tomography (CT) examinations were reconstructed using Advanced Workstation 4.4 (GE HealthCare, USA) for tumor volume measurement.

### Sample preparation and Sanger sequencing

Tumor and matched peripheral blood samples from 97 patients were obtained from the endocrine-related tumor bank of Shanghai Key Laboratory for Endocrine Tumors. The genomic DNA was prepared using the QIAGEN DNeasy blood and tissue kit (Qiagen, Hilden, Germany). Written informed consents were obtained from all of the study participants, and the protocols were approved by Ruijin Hospital Ethics Committee, Shanghai Jiao Tong University School of Medicine. The polymerase chain reaction (PCR) was performed in a Dual 96-well GeneAmp PCR system 9700 (Applied Biosystems, Courtaboeuf, France) using 20 ng of template DNA from each frozen sample per reaction. The products were sequenced by a 3730xl DNA analyzer (Applied Biosystems, Courtaboeuf, France). All of the sequences were analyzed using sequencing analysis software version 5.2 (Applied Biosystems, Courtaboeuf, France). The tumor samples had been screened for mutations in hotspot areas of *PRKACA* and *GNAS* and in the whole exomes of *PRKAR1A*.

### Immunohistochemistry for StAR

The adrenal glands were processed using immunohistochemistry as previously reported [19]. StAR immunostaining was performed on paraffin-embedded tissue samples from both the exploratory and confirmatory cohorts. The adrenocortical adenoma tissue specimens were cut and deparaffinized using standard histopathological procedures. The slides were then incubated overnight with the StAR monoclonal antibody (1:100, Cell Signaling Technology). Primary antibody binding was demonstrated using a standard avidin-biotin-peroxidase complex technique (Vector pk-7200). Diaminobenzidine was used to reveal the enzymatic activity, and the tissue sections were subsequently counterstained with hematoxylin. The cytoplasmic staining in the tumor cells was assessed as positive, and the H-score was used for semiquantitative analysis of StAR immunostaining. The investigators who reviewed the slides independently were blinded to the mutational status. The staining intensity was graded on the following scale: 0 (negative), 1 (weakly positive), 2 (positive), and 3 (strongly positive). The percentage score was multiplied by the staining intensity to obtain a final H-score, ranging from 0 to 300. We classified the samples into two groups based on the H-score: high and low expression (H-score ≥ 200 and = 100, respectively).

### Statistical analysis

The data are presented as proportions and the mean ± SD (for demographic data) or median (interquartile range). Continuous data were compared by using two-sample Student’s *t*-test or nonparametric tests, as appropriate. The categorical data were analyzed by using Fisher’s exact test. All of the statistical comparisons were two-sided, and *P* < 0.05 was considered statistically significant. The statistical analysis was performed using statistical package for the social sciences (SPSS) software version 18.0 (SPSS Inc., Chicago, IL, USA).

## Results

### Patient characteristics and endocrine evaluation

The cohort consisted of 97 unilateral cortisol-producing adenomas, including an exploratory cohort of 64 tumors and a confirmatory cohort of 33 tumors. The clinical characteristics of the cohort are summarized in Table 1. The mean age of the 97 patients was 39 years, and the cohort included 91 female and six male patients (ratio 15:2).

**Table 1:** Clinical, immunochemical, and genetic parameters of patients.

Adrenal cortisol-secreting adenomas	Exploratory cohort	Confirmatory cohort	Total
**Patients, n**	64	33	97
**Sex**			
Female (%)	61 (95.3)	30 (90.9)	91 (93.8)
Male (%)	3 (4.7)	3 (9.1)	6 (6.2)
**Age at diagnosis, yr**	38.6 ± 9.9	42.7 ± 13.7	39.9 ± 11.4
**High expression of StAR, n (%)**	48 (75.0)	24 (72.7)	72 (74.2)
**Mutational status**			
*PRKACA* mutation, n (%)	45 (70.3)	22 (66.7)	67 (69.1)
*GNAS* mutation, n (%)	2 (3.1)	3 (9.1)	5 (5.2)
*PRKAR1A* mutation, n (%)	1 (1.6)	0 (0)	1 (1.0)
**Tumor size from CT scan**			
Adrenal tumor volume, cm^3^	8.6 (6.5–11.8)	10.5 (7.7–13.2)	9.3 (6.8–12.5)
**Plasma cortisol, μg/dL**			
0800 h	20.9 ± 6.7	18.7 ± 4.3	20.2 ± 6.1
1200 h	18.7 ± 4.9	16.7 ± 3.1	18.1 ± 4.5
**UFC**, **μg/day**	395.4 (253.0–576.7)	429.2 (229.1–722.1)	399.1 (240.0–593.3)
**UFC per tumor volume, μg·cm**^**(3)-1**^**·day**^**1**^	42.2 (26.7–79.3)	40.2 (24.7–55.4)	41.1 (26.0–41.1)
**Plasma ACTH 0800 h, pg/mL**	9.1 (6.7–12.7)	7.5 (5.8–11.1)	8.3 (6.5–12.4)
**Plasma cortisol after LDDST, μg/dL**	21.6 (17.3–25.3)	18.8 (16.5–20.9)	20.8 (16.7–23.4)

StAR, steroidogenic acute regulatory protein; *PRKACA*, protein kinase cyclic adenosine monophosphate (cAMP)-activated catalytic subunit alpha; *GNAS*, guanine nucleotide binding protein, alpha stimulating; *PRKAR1A*, protein kinase cAMP-dependent type I regulatory subunit alpha; CT, computed tomography; UFC, urinary free cortisol; ACTH, adrenocorticotropic hormone; LDDST, low-dose dexamethasone test.

Data are presented as the mean ± standard deviation (SD) or median (interquartile range) for skewed variables or numbers (proportions) for categorical variables.

### StAR immunostaining in *PRKACA-*, *GNAS-*, and *PRKAR1A*-mutant and wild-type ACAs

**Fig 1:**
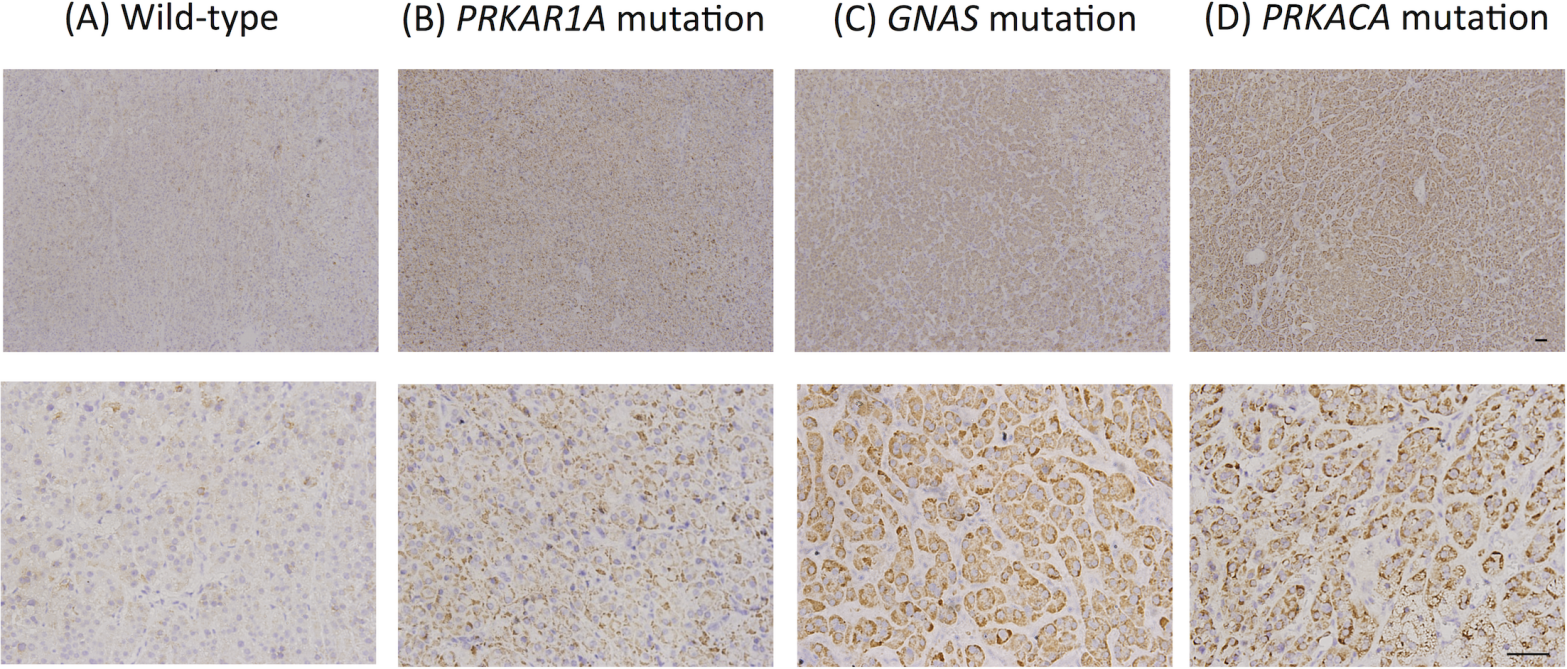
Immunohistochemical staining of steroidogenic acute regulatory protein (StAR) in cortisol-producing ACAs with different genotypes. (A) Wild-type adenoma presenting with low StAR expression (H-score = 100); adenomas with mutations in (B) *PRKAR1A* (H-score = 200), (C) *GNAS* (H-score = 200), and (D) *PRKACA* presenting with high StAR expression (H-score = 200) (scale bar, 50 μm). *PRKAR1A*, protein kinase cAMP-dependent type I regulatory subunit alpha; *GNAS*, guanine nucleotide binding protein, alpha stimulating; *PRKACA*, protein kinase cyclic adenosine monophosphate (cAMP)-activated catalytic subunit alpha.

The exploratory cohort (61 females and three males) with known mutational status [9] included 45 tumors with *PRKACA* mutations (70.3%), two with *GNAS* mutations, and one with a *PRKAR1A* mutation (Table 1). All of the *PRKACA* and *GNAS* mutations were L205R and R201 (R201H and R201C), respectively. We then explored the association between mutational status and StAR expression. Cytoplasmic localization of StAR immunostaining was observed in both wild-type and mutant adenoma tissues, whereas high expression was found in 75.0% (48/64) of the tumors. The cytoplasmic StAR immunostaining was more intense in tissues with mutations in *PRKACA*, *GNAS*, and *PRKAR1A* compared to the wild-type tumors (Fig 1), whereas StAR was not differentially expressed among the tissue samples with mutations in *PRKACA*, *GNAS*, and *PRKAR1A*. These indicate that high expression of StAR occurred in the mutated ACAs, and two distinct groups of tumors could be identified based on the differential expression of StAR.

We then analyzed the expression of StAR and association with genotype in the confirmatory cohort (n = 33, 30 female and three male patients) by performing Sanger sequencing. High expression of StAR was observed in 72.7% (24/33) of the ACAs. Mutations in *PRKACA* and *GNAS* were found in 66.7 and 9.1% (22/33 and 3/33) of the tumors, respectively. Furthermore, 87.5% (21/24) of the tumors showing high expression of StAR had the activating L205R mutation, 4.2% (1/24) had a non-frameshift deletion (c.731_745del/p.244_249 del) in the *PRKACA* gene, and 8.3% (2/24) had activating *GNAS* mutations (R201H and R201C). Only one tumor with low expression of StAR harbored the *GNAS* mutation R201H. Overall, immunohistochemical analysis revealed higher expression of StAR in adrenal tumors with mutations in *PRKACA*, *GNAS*, and *PRKAR1A* than in wild-type tumors, which exhibited lower expression of StAR.

In total, 93.1, 5.5, and 1.4% (67/72, 4/72, and 1/72) of the ACAs with high StAR expression carried mutations in *PRKACA*, *GNAS*, and *PRKAR1A*, respectively (Table 2). Additionally, 99% of adenomas bearing mutations in cAMP/PKA pathway-related genes (*PRKACA*, *GNAS*, or *PRKAR1A*) displayed high StAR expression.

**Table 2:** Clinical parameters and the mutational status (*PRKACA*, *GNAS*, and *PRKAR1A*) of cortisol-producing ACAs according to steroidogenic acute regulatory protein (StAR) immunohistochemistry.

Group	Immunohistochemistry of StAR		*P* value
	Low expression	High expression	
**Patient, n**	25	72	
**Sex**			
Female	22 (88.0)	69 (95.8)	0.18
Male	3 (12.0)	3 (4.2)	
**Age at diagnosis, yr**	40.3 ± 11.8	39.8 ± 11.3	0.85
**Mutational status**			
WT, n (%)	24 (96.0)	0 (0)	< 0.001
*PRKACA* mutation, n (%)	0 (0)	67 (93.1)	
*GNAS* mutation, n (%)	1 (4.0)	4 (5.5)	
*PRKAR1A* mutation, n (%)	0 (0)	1 (1.4)	
**Tumor size from CT scan**			
Adrenal tumor volume ^a^, cm^3^	13.2 (9.0–20.3)	8.3 (5.9–10.7)	< 0.001
**Plasma cortisol, μg/dL**			
0800 h	20.4 ± 6.3	20.1 ± 6.1	0.82
1200 h	18.2 ± 4.0	18.0 ± 4.7	0.85
**UFC**, **μg/d**	457.6 (344.6–622.0)	347.1 (226.5–591.2)	0.09
**UFC per tumor volume** ^**a**^**, μg/cm**^**3**^**/d**	35.3 (17.1–58.1)	44.0 (26.6–82.9)	0.032
**Plasma ACTH 0800 h, pg/mL**	8.2 (6.7–13.3)	8.4 (6.4–12.4)	0.78
**Plasma cortisol after LDDST, μg/dL**	20.2 (16.7–24.4)	20.8 (17.0–22.5)	0.35

StAR, steroidogenic acute regulatory protein; *PRKACA*, protein kinase cyclic adenosine monophosphate (cAMP)-activated catalytic subunit alpha; *GNAS*, guanine nucleotide binding protein, alpha stimulating; *PRKAR1A*, protein kinase cAMP-dependent type I regulatory subunit alpha; CT, computed tomography; UFC, urinary free cortisol; ACTH, adrenocorticotropic hormone; LDDST, low-dose dexamethasone test.

Data are presented as the mean ± standard deviation (SD) or median (interquartile range) for skewed variables and as numbers (proportions) for categorical variables. Skewed variables were logarithmically transformed before the analysis.

^a^ Values were adjusted for age and sex.

### Correlation of StAR expression with the clinical features of cortisol-producing adenomas

To determine the association between StAR expression and the clinical characteristics of the ACAs, we compared the biochemical and mutational features of ACAs with different degrees of StAR staining (Table 2). After adjusting for age and sex, the tumor volume of ACAs with high expression of StAR was significantly smaller than that of ACAs with low expression [8.3 (5.9–10.7) *vs*. 13.2 (9.0–20.3) cm^3^, *P* < 0.001]. Tumors with high expression of StAR also had significantly higher 24-h urinary free cortisol levels per tumor volume than those with low expression did [44.0 (26.6–82.9) *vs*. 35.3 (17.1–58.1) μg/cm^3^/day; *P* = 0.032]. Smaller adenomas and increased cortisol production were more prevalent in ACAs with high StAR expression than in those with low StAR expression. No significant differences were observed in sex, age at diagnosis, midnight cortisol, urinary free cortisol, ACTH levels, and plasma cortisol after LDDST between groups with high and low expression of StAR.

## Discussion

In our study, high expression of StAR was observed in the majority (74.2%) of cortisol-producing adenomas. StAR was overexpressed in all ACAs with mutations in *PRKACA*, whereas it was clearly expressed at lower levels in wild-type tumors. Furthermore, high expression of StAR was observed in the ACAs with mutations in *GNAS* and *PRKAR1A* as a result of activation of cAMP/PKA signaling. Only one tumor with a *GNAS* mutation showed low expression of StAR, possibility because of the procedures used to prepare the formalin-fixed paraffin embedded (FFPE) samples. No difference in StAR staining was observed among adenomas with mutations in *PRKACA*, *GNAS*, and *PRKAR1A*. We included five ACAs with *GNAS* mutations and one with a *PRKAR1A* mutation in our study; more patients are needed to adequately compare StAR expression among the three mutant groups in future studies.

StAR expression demonstrated a clear correlation with cAMP/PKA pathway-related gene mutations. Our previous study showed that PKA activity and StAR expression were significantly increased in ACAs with mutations in *PRKACA* [9]. The induction of cAMP and the resulting cAMP-dependent signaling is undoubtedly the principal pathway that regulates StAR expression and steroid biosynthesis. Furthermore, StAR can be directly activated by PKA phosphorylation. The present study demonstrated high expression of StAR in tumors with mutations in *PRKACA*, *GNAS*, and *PRKAR1A*. This finding may support a role for StAR in the activation of cAMP/PKA signaling, which is consistent with a previous study that showed increased staining of StAR in primary pigmented nodular adrenocortical disease (PPNAD) compared to control tissues [20]. PKA activation induces phosphorylation of CREB, which initiates transcription of StAR; therefore, StAR might be regarded as a marker for activation of cAMP/PKA signaling.

Our results show that StAR immunostaining, as a convenient, rapid, and applicable alternative strategy, may be used in cortisol-producing ACAs to determine whether activation of cAMP/PKA signaling is present. Furthermore, classification of tumors based on the expression of StAR might be representative of the clinical features of ACAs. Steroid hormone biosynthesis in steroidogenic cells is primarily regulated by activation of cAMP/PKA signaling [21,22], and tumors with different mutated genes might present similar clinical parameters. Adrenal adenomas harboring mutations in *PRKACA* or *GNAS* are generally smaller [10,11,13] and present with more overt hypercortisolism [8,10,13] than the non-mutant types. Patients with tumors carrying mutations in *PRKACA* generally present with the disease at younger ages than those without mutations [11]. In the present study, a more severe phenotype was identified in tumors with high expression of StAR than in those with low expression. Furthermore, the ACAs could be categorized into two groups (high and low expression of StAR) based on the pathological classification. ACAs having high expression of StAR exhibited higher cortisol secretion per tumor volume (*P* = 0.032) and smaller tumor volume (*P* < 0.001) than those with low expression. However, no significant differences in urinary and midnight cortisol or in cortisol after LDDST were observed in the current study. Patients with adrenal Cushing’s syndrome were predominantly female. Thus, the female dominance in the occurrence of *PRKACA*-associated tumors may be linked to this condition; these aspects require further investigations. Furthermore, the particular clinical parameters reported in previous studies were not completely consistent [8–13,23]. The results could have been confounded by the variability in the clinical recognition of the cortisol-associated phenotypes or image findings.

Abnormal cytoplasmic StAR immunohistochemical staining was evident in all ACAs with PKA-activating mutations, which suggests that cortisol-producing ACAs can be classified into two categories based on StAR immunostaining: PKA-activating and normal PKA activity. The tumors with high and low expression of StAR were PKA-activating and normal PKA activity types, respectively. The PKA-activating ACAs had generally prior stronger cortisol secretory capacity more than tumor growth.

In conclusion, this study demonstrated the association between high expression of StAR and PKA activation in ACAs, which further implied that StAR immunohistochemistry could be a valuable tool for identifying cortisol-producing adenomas that activate the cAMP/PKA signaling pathway. The finding provides a potential novel marker for the molecular pathological diagnosis of PKA-activating ACAs.
